# Supplementary material for: Long-distance tmFRET using bipyridyl- and phenanthroline-based ligands
Source: bioRxiv. 2023 Oct 12:2023.10.09.561591. Preprint. [Version 1] doi: 10.1101/2023.10.09.561591 (PMC10592757; doi:10.1101/2023.10.09.561591)
Supplement: Supplementary file 1 [file NIHPP2023.10.09.561591v1-supplement-1.pdf]

bioRxiv preprint doi: https://doi.org/10.1101/2023.10.09.561591; this version posted October 12, 2023. The copyright holder for this preprint (which was not certified by peer review) is the author/funder, who has granted bioRxiv a license to display the preprint in perpetuity. It is made available under aCC-BY 4.0 International license.

SUPPLEMENTAL FIGURE LEGENDS

Figure S1

Using Acd and $[Cu(TETAC)]^{2+}$ to measure maltose-dependent change in distance on the lip of MBP clamshell. (**A,B**) Time courses of quenching by Cu^{2+}-cyclenM in the absence and presence of maltose, as indicated above each graph. The donor and acceptor site for each experiment are shown above each graph. The number of experimental replicates is listed on the scatter plots in (C) and (D). (**C,D**) Collected data for each of the graphs shown in (A) and (B) measured at steady state.

Figure S2

Whole protein mass spectra comparing cyclen chelator labeling strategies. (**A**) Mass spectra of MBP-295Acd-237C before and after labeling with 10 and 40 µM $[Cu(TETAC)]^{2+}$. The 40 µM $[Cu(TETAC)]^{2+}$ spectrum is vertically offset for clarity. (**B**) Mass spectra of MBP-295Acd-237C before and after labeling with 80 µM cyclen maleimide. The Δ18 peak seen in the labeled sample comes from the hydrolysis of the maleimide ring.

Figure S3

Whole protein mass spectra confirm labeling with metal chelators. (**A**) Mass spectra of MBP-322Acd-278 before and after labeling with $[Ru(bpy)_2phenM]^{2+}$. (**B**) Mass spectra of MBP-295Acd-211C before and after labeling with phenM. The δ18 peak seen in both labeled samples comes from the hydrolysis of the maleimide ring.

Figure S4

Strategies for labeling with (**A**) 1:3 Fe^{2+}:phenanthroline and (**B**) 1:1 Fe^{2+}:phenanthroline.

Figure S5

Quenching of Acd in MBP by DabcylM. Time courses of quenching of (**A**) MBP-295Acd-237C; (**B**) MBP-322Acd-309C; (**C**) MBP-295Acd-211C; and (**D**) MBP-322Acd-278C. The number of replicates for each experiment is shown in (E). (**E**) Collected data for each of the graphs shown in (A-D) measured at steady state.

bioRxiv preprint doi: https://doi.org/10.1101/2023.10.09.561591; this version posted October 12, 2023. The copyright holder for this preprint (which was not certified by peer review) is the author/funder, who has granted bioRxiv a license to display the preprint in perpetuity. It is made available under aCC-BY 4.0 International license.

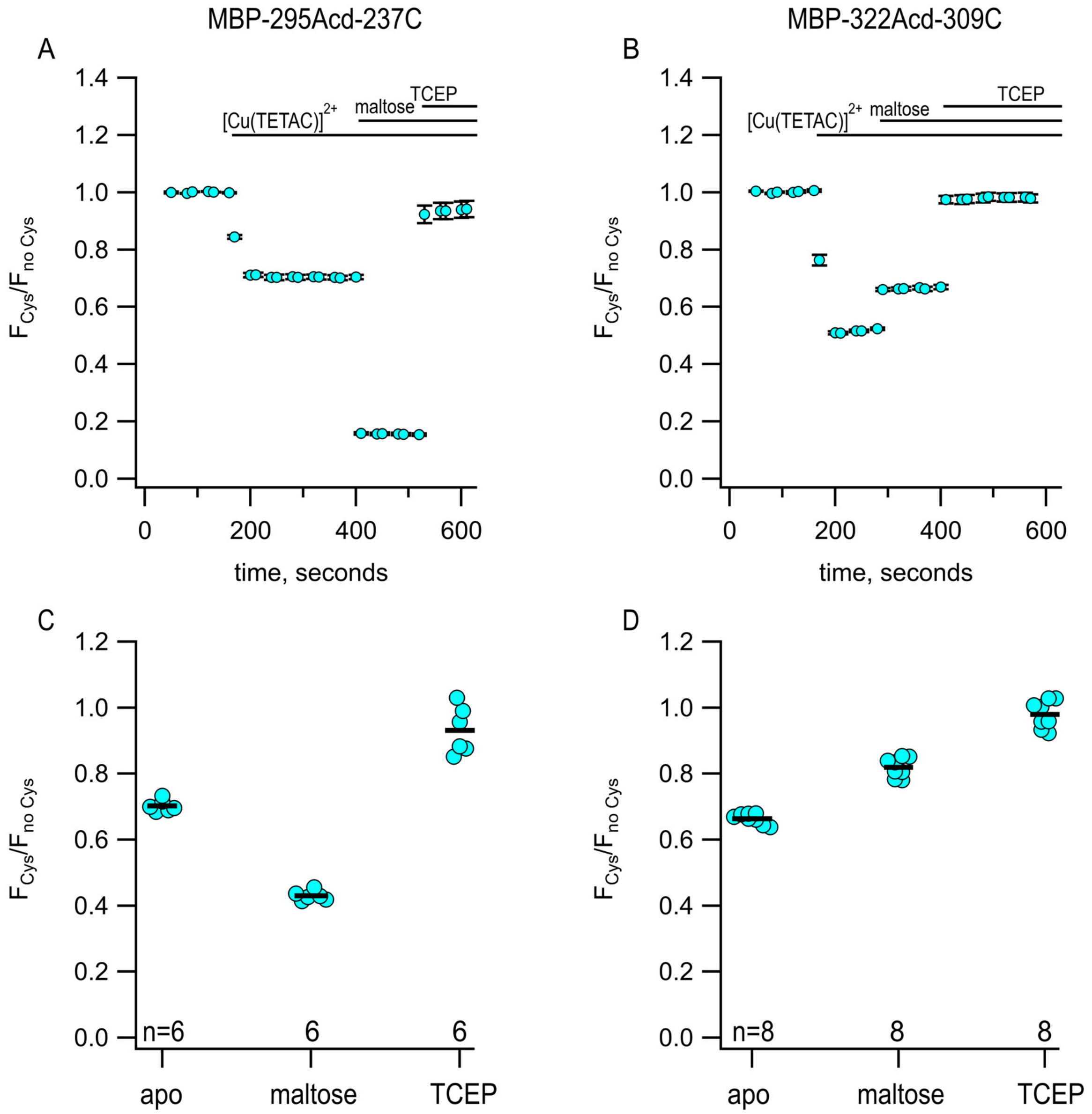

Figure S1

bioRxiv preprint doi: https://doi.org/10.1101/2023.10.09.561591; this version posted October 12, 2023. The copyright holder for this preprint (which was not certified by peer review) is the author/funder, who has granted bioRxiv a license to display the preprint in perpetuity. It is made available under aCC-BY 4.0 International license.

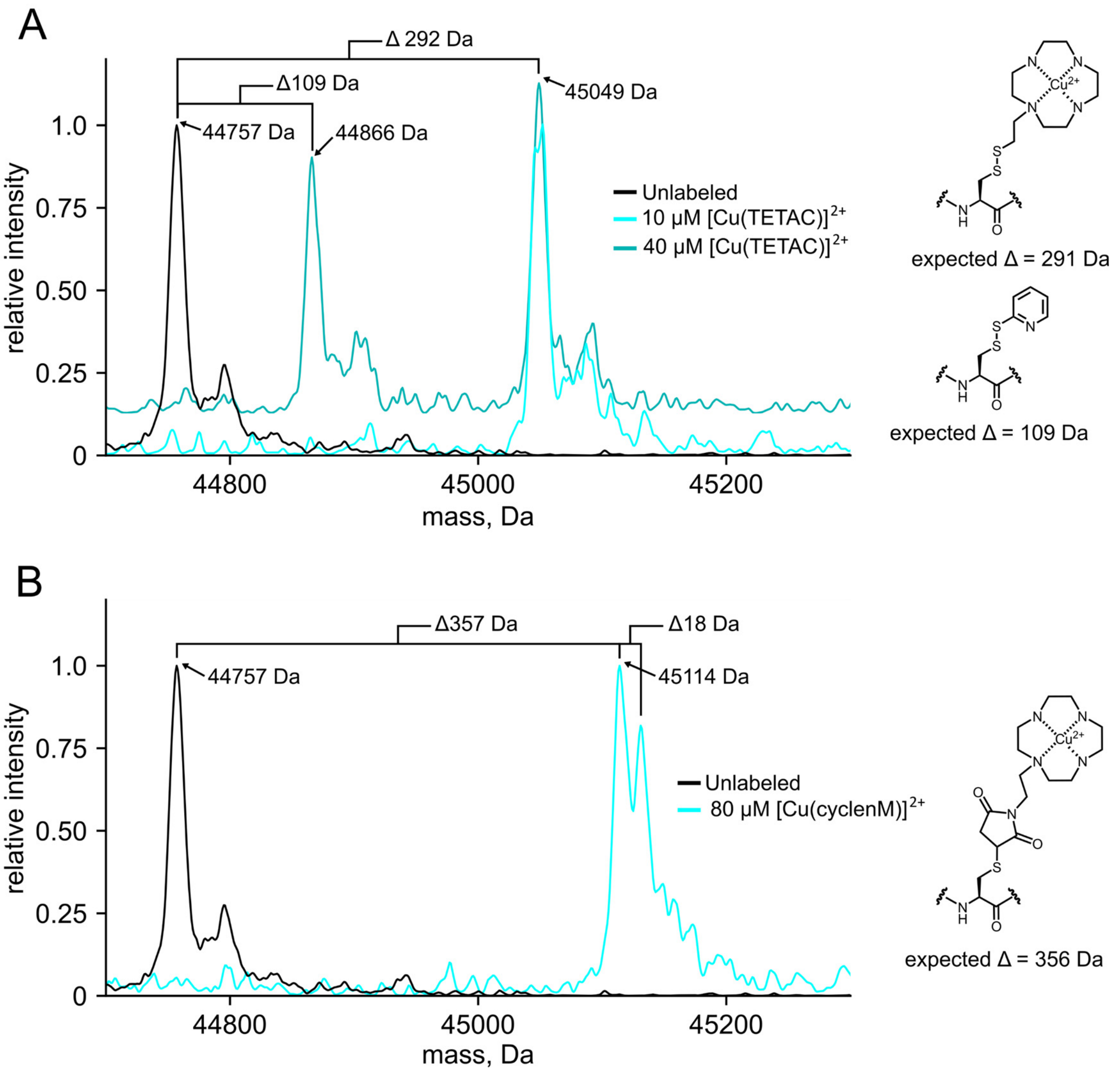

Figure S2

bioRxiv preprint doi: https://doi.org/10.1101/2023.10.09.561591; this version posted October 12, 2023. The copyright holder for this preprint (which was not certified by peer review) is the author/funder, who has granted bioRxiv a license to display the preprint in perpetuity. It is made available under aCC-BY 4.0 International license.

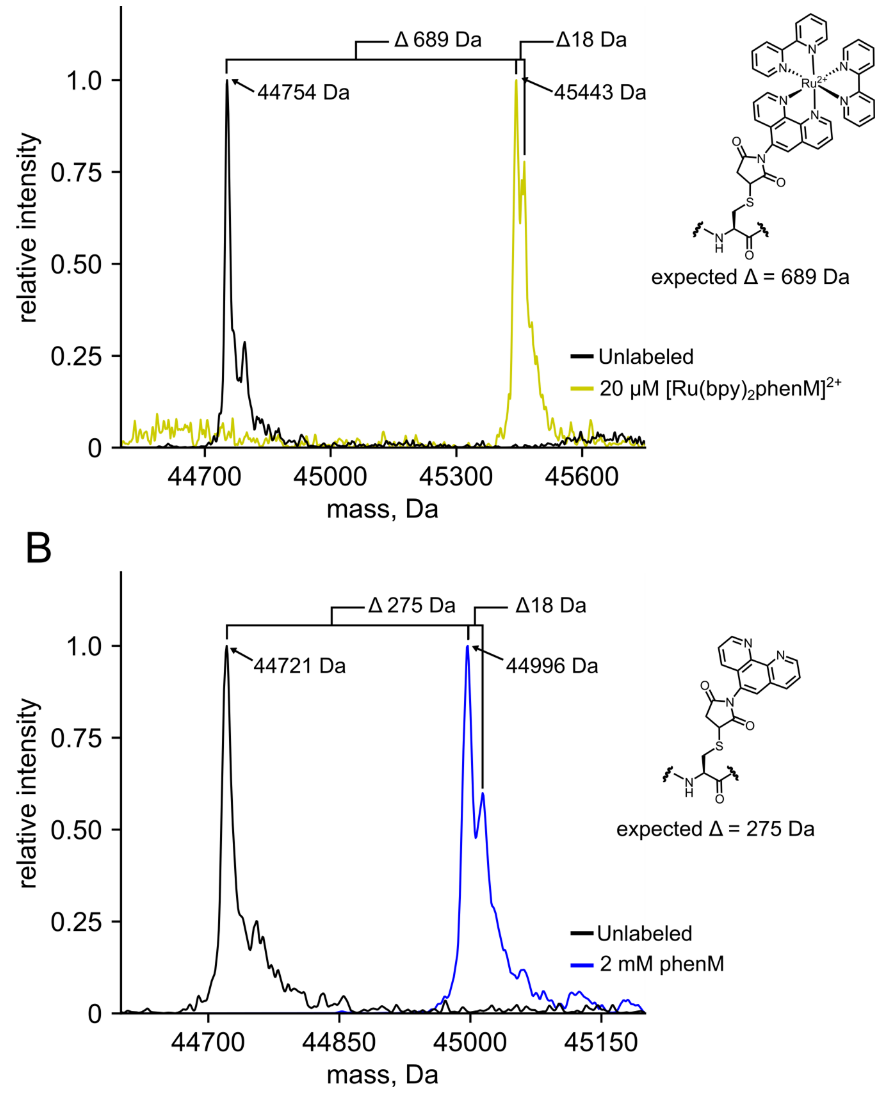

Figure S3

bioRxiv preprint doi: https://doi.org/10.1101/2023.10.09.561591; this version posted October 12, 2023. The copyright holder for this preprint (which was not certified by peer review) is the author/funder, who has granted bioRxiv a license to display the preprint in perpetuity. It is made available under aCC-BY 4.0 International license.

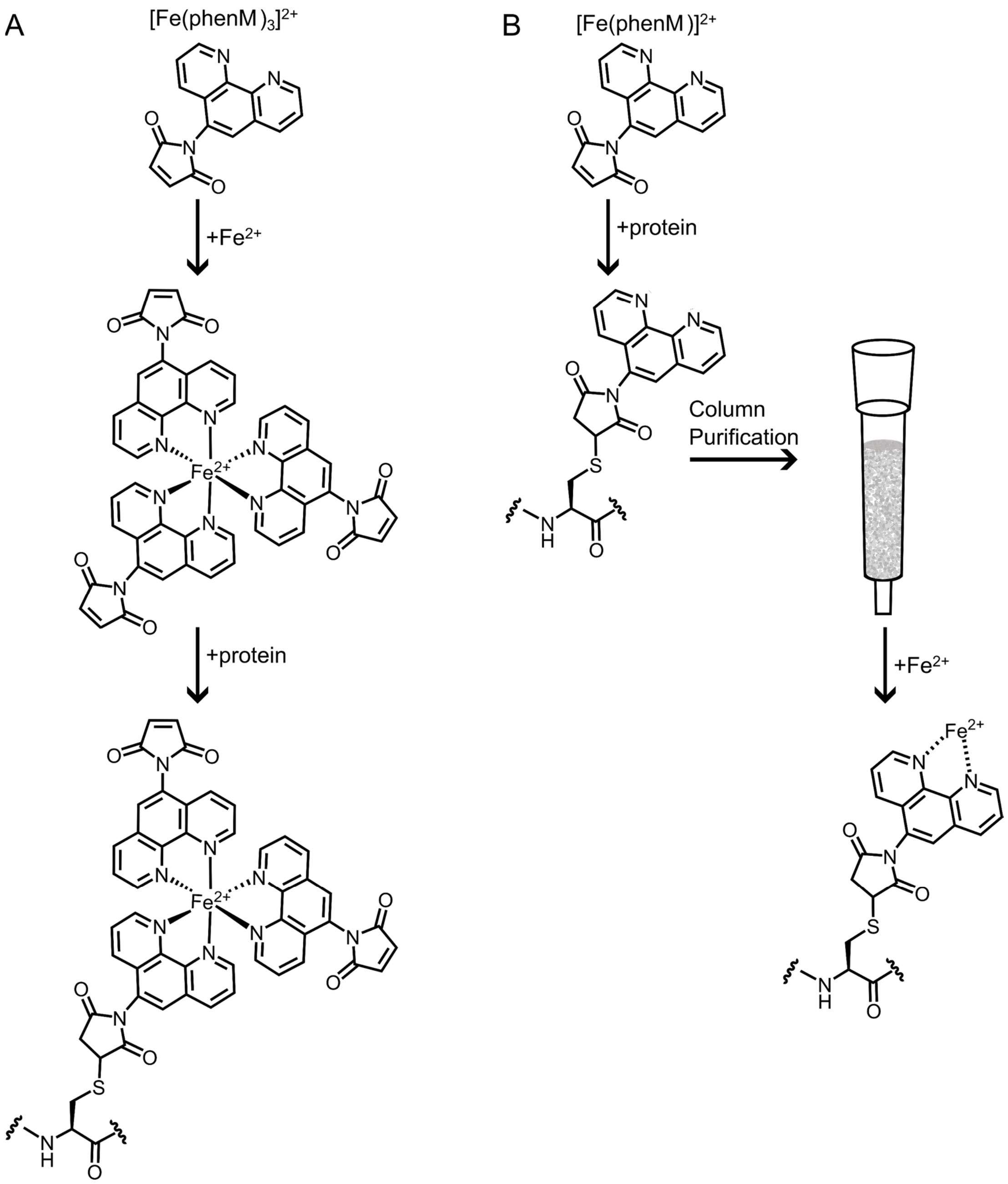

Figure S4

bioRxiv preprint doi: https://doi.org/10.1101/2023.10.09.561591; this version posted October 12, 2023. The copyright holder for this preprint (which was not certified by peer review) is the author/funder, who has granted bioRxiv a license to display the preprint in perpetuity. It is made available under aCC-BY 4.0 International license.

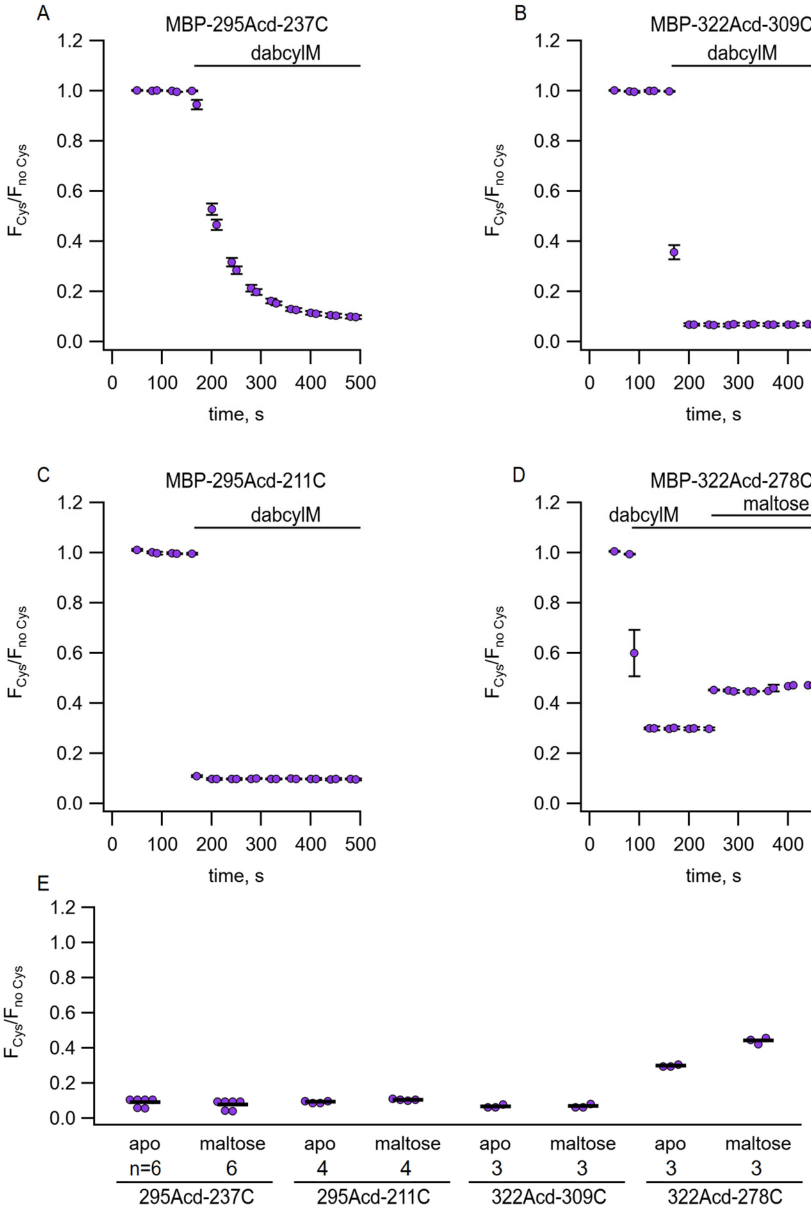

Figure S5
